# Accuracy Evaluation of 14 Maxillary Full Arch Implant Treatments Performed with Da Vinci Bridge: A Case Series

**DOI:** 10.3390/ma13122806

**Published:** 2020-06-22

**Authors:** Luigi V. Stefanelli, George A. Mandelaris, Alessio Franchina, Dario Di Nardo, Massimo Galli, Michele Pagliarulo, Luca Testarelli, Stefano Di Carlo, Gianluca Gambarini

**Affiliations:** 1 Department of Oral and Maxillo-Facial Sciences, Sapienza University of Rome, 00161 Rome, Italy; gigistef@libero.it (L.V.S.); massimo.galli@uniroma1.it (M.G.); luca.testarelli@uniroma1.it (L.T.); stefano.dicarlo@uniroma1.it (S.D.C.); gianluca.gambarini@uniroma1.it (G.G.); 2 Private Practice, Periodontics and Dental Implant Surgery; Periodontal Medicine & Surgical Specialists, LTD, Chicago, IL 60601, USA; gmandelaris@periodontalmedicine.org; 3 Private Practice, Periodontics and Dental Implant Surgery, 36100 Vicenza, Italy; alessiofranchina@icloud.com; 4 Faculty of Dental Medicine, University of Plovdiv, 4002 Plovdiv, Bulgary; michele.pagliarulo2000@gmail.com

**Keywords:** dynamic navigation implantology, pterygoid implants, atrophic maxilla, totally edentulous patients, computer aided implantology

## Abstract

The use of pterygoid implants can be an attractive alternative to sinus bone grafting in the treatment of posterior atrophic maxilla. This technique has not been widely used because of the difficulty of the surgical access, the presence of vital structures, and the prosthetic challenges. The use of dynamic computer aided implantology (DCAI) allows the clinician to utilize navigation dental implant surgery, which allows the surgeon to follow the osteotomy site and implant positioning in real time. A total of 14 patients (28 pterygoid implants and 56 intersinusal implants) were enrolled in the study for a full arch implant prosthetic rehabilitation (4 frontal implants and 2 pterygoids implants), using a dynamic navigation system. The reported accuracy of pterygoid implants inserted using DCAI was 0.72 mm at coronal point, 1.25 mm at apical 3D, 0.66 mm at apical depth, and 2.86° as angular deviation. The use of pterygoid implants in lieu of bone grafting represents a valid treatment opportunity to carry out a safe, accurate, and minimally invasive surgery, while reducing treatment time and avoiding cantilevers for a full implant prosthetic rehabilitation of the upper arch.

## 1. Introduction

Implant–prosthetic rehabilitations have greatly improved the quality of life for partially and fully edentulous patients [1,2,3].

Cumulative survival rates of osseointegrated implants in totally edentulous patients over a 10-year observational period have been shown to exceed 90%. However, placing an implant in the posterior atrophic maxilla is a challenge because of bone availability and poor bone quality, and there are also challenges associated with the difficulty of the surgical access [4,5,6].

The insertion of an implant in a poor bone quality has been demonstrated to have a higher rate of failure. [7] In addition, implant length affects success rate when the implant is inserted in Type III and Type IV bone quality. On the other hand, the length of an implant seems to have less importance on stress distribution, except for instances where Type IV bone quality exists where the implant length plays a key role [8,9,10,11].

Several techniques have been proposed for the treatment of the totally edentulous atrophic upper jaw: use of tilted implants, sinus floor elevation, short and ultra-short implants, and pterygoid or zygomatic implants [12,13,14,15,16,17,18,19,20,21,22,23].

The use of pterygoid implants was proposed for the first time by Tulasne et al. in 1989 as an implant to be inserted through three different bones (maxillary tuberosity, horizontal process of the palatine bone, and pterygoid process of the sphenoid bone) engaging the pterygoid plate. In this area, 80% of the native bone remains stable over time, even after atrophy and residual ridge resorption patterns ensue following extractions [24,25].

Inserting a pterygoid implant with DCAI allows several advantages [24,25,26,27,28,29,30]:

- Minimally invasive (no bone grafting) approach;
- Avoid posterior prosthetic cantilevers;
- Predictability;
- Shorter period of treatment;
- Stable during the time;
- Immediate loading is possible;
- Decreasing of costs (patient and dentist).

In this direction, the use of pterygoid implants could be a suitable alternative to a sinus bone graft in the treatment of posterior atrophic maxilla [24,28,29,30].

Notwithstanding the use of pterygoid implant is a reasonable option, this technique has not been widely used because of the difficulty of surgical access in this area (insertion of a long implant with a semi-blinded surgery made free-hand), the presence of vital structures (greater palatine artery and pterygomaxillary fossa), and the prosthetic difficulty in the short- (tilted implants in a limited vertical space of this region) and long-term (owing to high occlusal masticatory stresses in this area that could end in prosthesis or screw fractures) [24,25,26,27,28,29,30].

For the above mentioned reasons, the accurate placement of pterygoid implants (13–20 mm of implant length) using free-hand techniques becomes incredibly difficult compared with the other proposed techniques for the rehabilitations of atrophic edentulous maxilla [26,27,28,29,30].

The use of computer-aided implantology (CAI) has been reported to be accurate by measure of minimum deviations between planned and inserted implants [31,32,33].

In particular, the use of dynamic computer-aided implantology (DCAI) allows the clinician not only to be computer guided during surgery (following in real time the advancing of the drill into the bone), but also to verify (by touching a landmark) if the workflow is progressing in an accurate way; the latter option becoming very important in such high-risk anatomic areas.

A new workflow involving dynamic navigation, called Trace and Place (TaP), has been enabled by ClaroNav™, a Canadian company manufacturing Navident^®^, in which patient’s jaw registration to her cone beam computerized tomography (CBCT) images is accomplished by tracing the existing teeth or mini screws, both of which can be used as fiducial markers [31,32,33,34], thus performing dynamic guided surgery without any in-mouth template. This provides a fully digital implant–prosthetic workflow and is less obtrusive [35,36].

Furthermore, the use of angled multi-unit abutment (M.U.A) can reduce prosthodontic difficulties by allowing tilted implant axis correction.

Another solution for the final prosthesis is the OT Equator™ (Rhein83 Srl, Bologna, Italy) implant connection. It comes from the Normo Ball attachment concept and can be used in both fixed and removable prostheses.

When used in place of an M.U.A. for fixed screw-retained dentures, it allows the OT Bridge™ turrets to compensate up to 80° of divergence between two implants as a result of its “Extragrade™” feature. “Extragrade” is a minimal flap that, if correctly positioned, allows the metal structure to overcome an implant created undercut and gets connected with OT Equator in a passive manner.

The Seeger (Rhein83 Srl, Bologna, Italy) inserted inside the OT Bridge turrets engages the undercuts of the OT Equator head, holding the structure passively anchored. To complete, the screw is inserted onto OT Equator’s head.

The OT Bridge prosthetic screw has a core of 1.3 mm compared with the 1 mm standard M.U.A. screw core. This allows an advantage in terms of toughness and resistance to mechanical stresses and strains (resistance to stresses is directly proportional to section).

The aim of this study is to evaluate the placement accuracy of both pterygoid and frontal implants for a full maxillary implant supported rehabilitation.

The second aim of this study is to suggest a new terminology for this implant layout named as a Da Vinci bridge.

## 2. Material and Methods

### 2.1. Study Design

This study is a clinical single blinded retrospective case series.

### 2.2. Study Population/Demographics

Fourteen patients treated between 1 February 2018 and 30 September 2019 for a full arch implant supported rehabilitation were included in this study. All the treatments followed the concept of the “Da Vinci Bridge” and were performed at Department of Periodontics and Implant Dentistry at the Policlinico Umberto I, Sapienza University of Rome, Italy.

The “Da Vinci bridge” is a concept suggested in this paper to identify the placement of two to four implants in the frontal area between the two anterior maxillary sinus walls and two implants in the pterygoid process area [24,25,26,27,28,29,30].

Written informed consent was obtained from each patient after a detailed description of the study protocol. The research protocol was in accordance with “1975 Declaration of Helsinki” on medical protocols and ethics and its later amendments. A post-operative CBCT scan was included to assess the accuracy of implant placement related to virtual plan. This study protocol was approved by the Department of Oral and Maxillofacial Sciences—Sapienza, University of Rome (protocol identifying number: 582/17).

### 2.3. Inclusion and Exclusion Criteria

The CBCTs used for the retrospective study were selected from a pool of clinical cases complying with the following criteria at the time of surgery:

#### 2.3.1. Inclusion Criteria

- Any totally or partially edentulous patient that needs a full arch implant prosthetic rehabilitation.
- Bone crest width and height in the area between right and left premolars enough to place two to four implants with at least 1 mm of bone around the implant.
- Bone width and height in the pterygoid area enough to place one implant at least 13 mm long per side.

#### 2.3.2. Exclusion Criteria

- Patients with general contraindications to implant surgery.
- Patients with systemic diseases that could influence the outcome of the therapy (i.e., diabetes with HbA1c ≥ 6.5%, osteoporosis, or use of bisphosphonate medications).
- Patients with a history of radiation to the head and neck region.
- Patients who are pregnant or nursing.
- Patients with no available bone to plan a pterygoid implant.
- Patients with residual bone in the molar area higher and wider than 6 mm.
- Patients with insufficient residual bone crest in the frontal area to place two to four implants.

### 2.4. Trace and Place (TaP™) Protocol

The workflow has been described in previous publications [32,34], but is described here briefly. TaP protocol consists of three steps: (1) Plan: creation of a virtual surgical plan on the basis of the volumetric Digital Imaging Communication in Medicine (DICOM) data acquired from a cone beam computerized tomography (CBCT) scan. (2) Trace: registration of the patient’s jaw to CBCT. This is done by tracing radiopaque landmarks that get selected/marked on patient’s CBCT. (3) Place: navigated implant placement according to the plan.

#### 2.4.1. Plan

**Figure 1:**
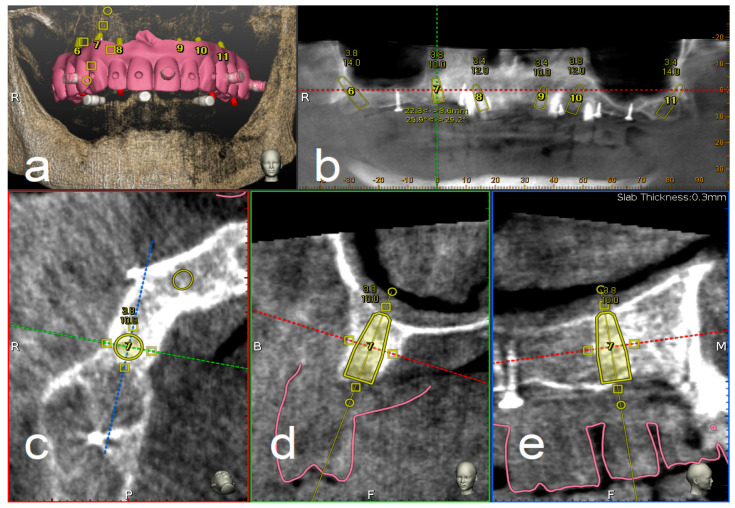
Implant planning using stereolithography (STL) files as reference for a prosthetic driven implantation (**a**). Panoramic (**b**), axial (**c**), bucco-lingual (**d**), and parasagittal (**e**) view.

A CBCT (Scanora 3Dx) and an intraoral surface scan (IOS) were taken on each patient. An ideal virtual wax-up of teeth was completed by Lab Technician. Both DICOM files from CBCT and stereolithography (STL) files from the IOS were matched in Navident software and semi-automatically superimposed to residual teeth (or in toothless cases, using reference points in the wax-up) using the provided mesh-to-image registration tool. In addition, the STL files of the final teeth set-up were matched above previous IOS files of the baseline oral conditions and displayed in Navident software to perform prosthetically driven implant planning (Figure 1).

#### 2.4.2. Trace

**Figure 2:**
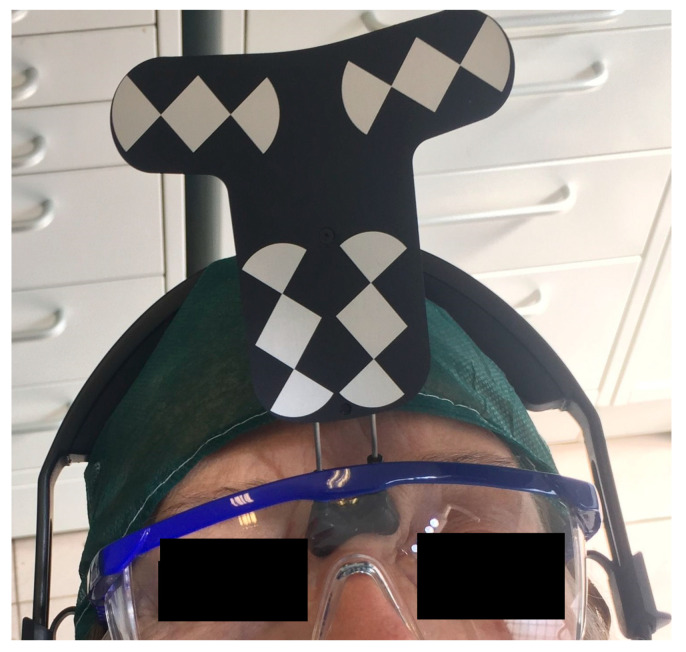
Head tracker used in the upper jaw for dynamic navigation Trace and Place (TaP).

**Figure 3:**
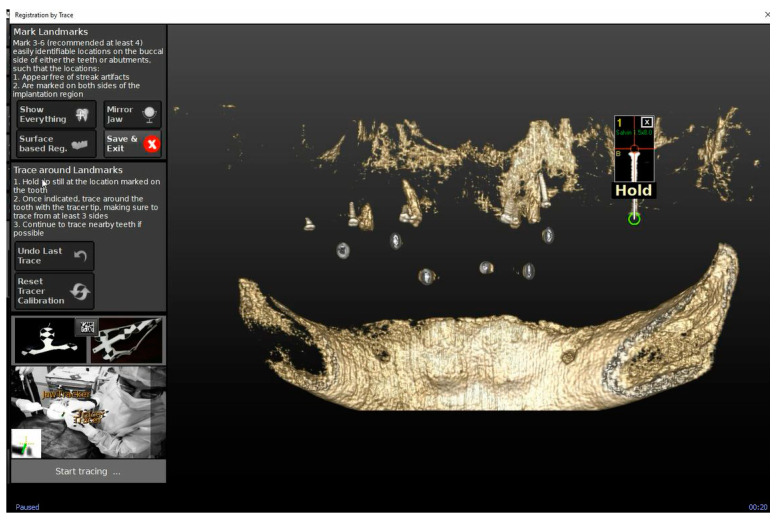
The figure shows the mini screws automatic recognition by the software and the related tracing progress.

To track the patient’s jaw by the system cameras, an optical tracking tag needs to be fixed to the patient’s jaw where the surgery has to be performed. This requires a JawTracker™ to be connected to 1–2 teeth in the patient residual dentition by light-curing composite resin or by anchoring with mini screws into bone. Alternatively, but only in the maxilla, a HeadTracker™ can be used for tracking by placing it directly on the patient’s head (Figure 2). Tracing can then be performed starting at landmark locations. During tracing, the surgeon slides the tracer’s ball tip in full contact with each landmark surface (if Salvin™ mini screws are used, software automatically recognized them once they come into contact with the tracer) (Figure 3).

**Figure 4:**
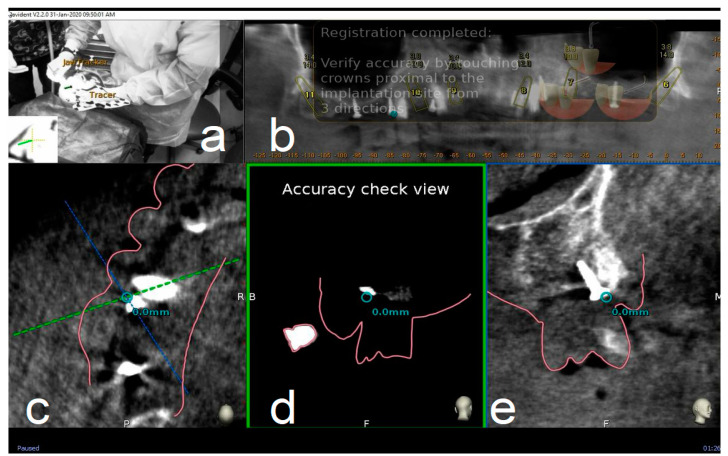
The surgeon (**a**) can then verify the registration accuracy (**b**) by touching with the tracer’s ball tip one of the patient’s landmark (mini screw used in this case) (**c**). The matching quality can be verified from each view (**d**,**e**).

After all selected landmarks/teeth are traced, the software automatically performs registration. Sampled trace points get superimposed with CBCT 3D rendering. The complete trace and registration process takes an average of 1–2 min. The accuracy of trace registration is then assessed by touching with tracer’s ball tip any patient’s anatomical marker and confirming congruency between the touched marker and what is shown on the laptop screen (Figure 4). If the accuracy check is not satisfactory, the tracing process can be immediately repeated.

#### 2.4.3. Place

**Figure 5:**
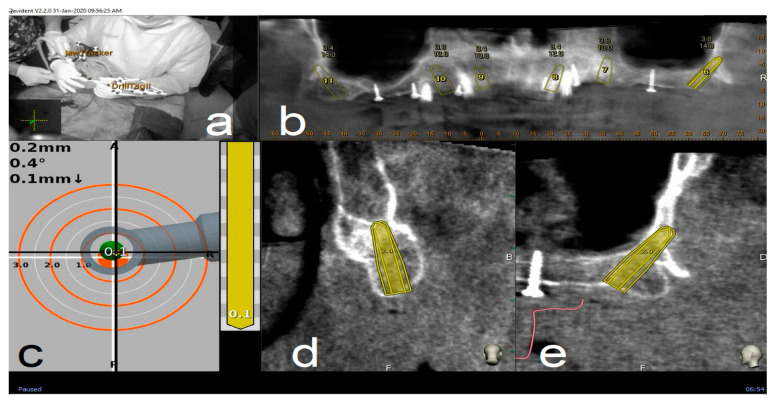
Clockwise from up left. The figure indicates the several views on the screen during surgery: tracker video stream (**a**), panoramic view (**b**), target view and depth indicator (**c**), bucco-lingual section view (**d**), and mesio-distal section view (**e**).

Handpiece drill axis and drill tip length are then calibrated using a metallic caliber; a second accuracy check is carried out in the same manner as for tracing. Once accuracy is confirmed, navigated implant placement can be carried out following target view. This allows clinician to verify, in real time, entry point, depth, and angulation of planned osteotomy as related to the plan. Other views that the clinician can see on the screen enable her/him to follow the position of the handpiece drill during osteotomy in coronal and sagittal views (Figure 5).

### 2.5. Surgical Treatment

Before the surgical treatment, all patients underwent an oral hygiene protocol that consisted of supra- and subgingival debridement and final polishing. One hour prior to surgery, patients received prophylactic antibiotic therapy with 2 g of Amoxicillina (GlaxoSmithKline, London, UK). Immediately before the procedure, they were instructed to rinse with a 0.2% chlorhexidine digluconate solution (Corsodyl, GlaxoSmithKline Consumer Healthcare, Genval, Belgium) for 2 min. All surgical procedures were performed by same experienced surgeon (L.V.S.). Local anesthesia was obtained with 2% mepivacaine 1:100,000 adrenalin (Carbocaine, AstraZeneca, Milan, Italy).

Four frontal implants and two pterygoid implants (TPA, AZ implant, San Lazzaro di Savena, Bologna, Italy), for a total of six implants (Da Vinci Bridge^®^), were planned and inserted in each upper arch.

For partially edentulous patients, the first inserted implants were those planned in healed ridges. Then, immediate implants were placed and, finally, all residual teeth were extracted.

After implant insertion and/or teeth extraction, multi-unit abutments (or alternatively OT Equator™ abutments) were screwed and an impression was taken to prepare a provisional screwed prosthesis.

The healing screws were screwed at 20 Ncm torque value.

On the basis of the implant angulation degree, an MUA or OT Equator abutment was selected in order to obtain a passive fit of the temporary prosthesis.

**Figure 6:**
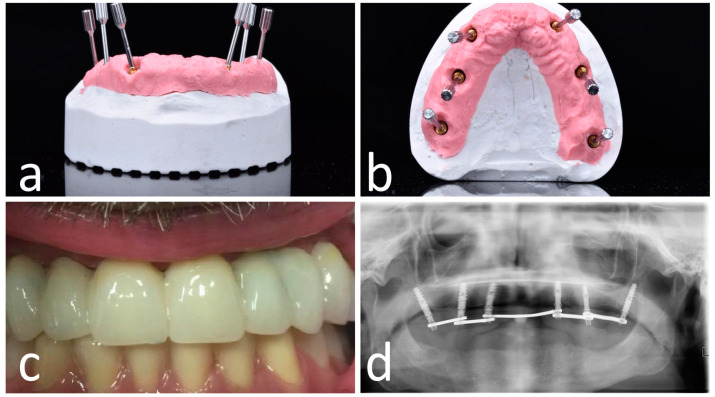
OT-bridge abutments use the “Extragrade™” feature, a system allowing to compensate up to 80° of the divergence between two implants (**a**,**b**). Pictures c and d show the clinical (**c**) and X-ray (**d**) view of the provisional prosthesis using OT-bridge abutments.

**Figure 7:**
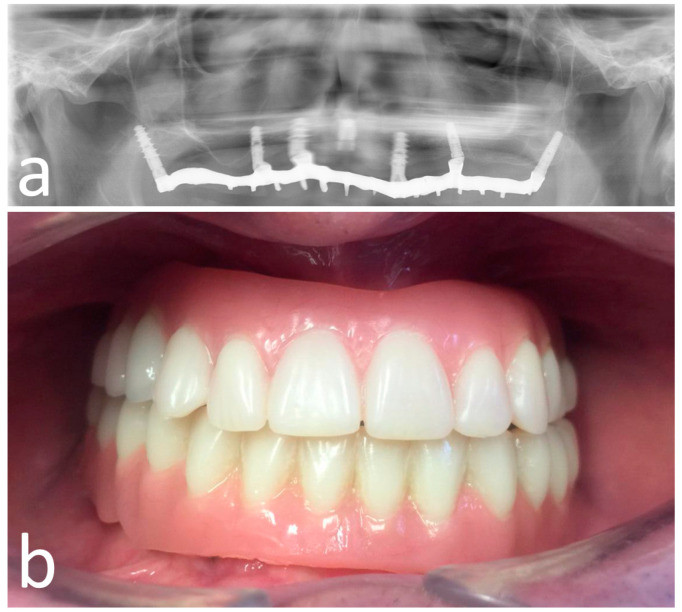
Opt X-ray (**a**) and clinical (**b**) view of the provisional prosthesis realized using conventional straight and angled multi-unit abutment (M.U.A.).

After 6 h, the provisional prosthesis was screwed, using the OT-bridge (Figure 6) or conventional M.U. abutments (Figure 7).

### 2.6. Post-Surgical Protocol

All patients were prescribed Augmentin (GlaxoSmithKline, London, UK) 1 g twice daily for 7 days. After surgery, analgesia was achieved with 200 mg of ketoprofen (Ibifen, Aprilia, Latina, Italy) for a maximum of three times daily according to the needs of individual patients. Each patient was instructed to rinse with 0.12% chlorhexidine digluconate (Corsodyl, GlaxoSmithKline Consumer Healthcare) three times daily for 2 weeks; to follow a soft diet for 1 week; and to gently cleanse with a soft toothbrush, avoiding the use of floss in the surgical area for the first month post-operatively.

### 2.7. Outcome Measures

#### 2.7.1. Post-Surgery Complications

The possible following post-surgery complications were recorded: (1) early implant failure, (2) post-operative infection, and (3) post-operative hemorrhage.

#### 2.7.2. Accuracy Evaluation Assessment

The assessment of accuracy was determined by overlapping the pre-operative CBCT with planned implants and the post-operative CBCT with achieved implants. This analysis was performed by two independently calibrated investigators (A.F., S.D.C.) who were blinded to other aspects of the study.

Any disagreement was solved by consensus, and a third investigator was consulted when it was not initially possible to achieve complete agreement (defined as the difference between the measurements made by the two experts of >0.1 mm).

**Figure 8:**
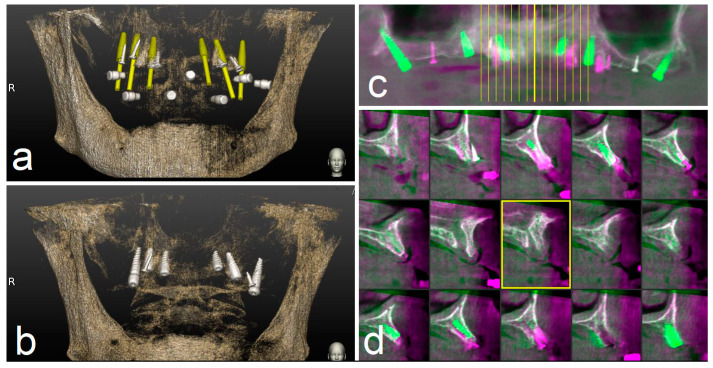
The preoperative surgical plan (**a**) and the postoperative cone beam computerized tomography (CBCT) (**b**) were superimposed using accuracy evaluation software (**c**,**d**). The registration was performed directly between the two volumetric images.

**Figure 9:**
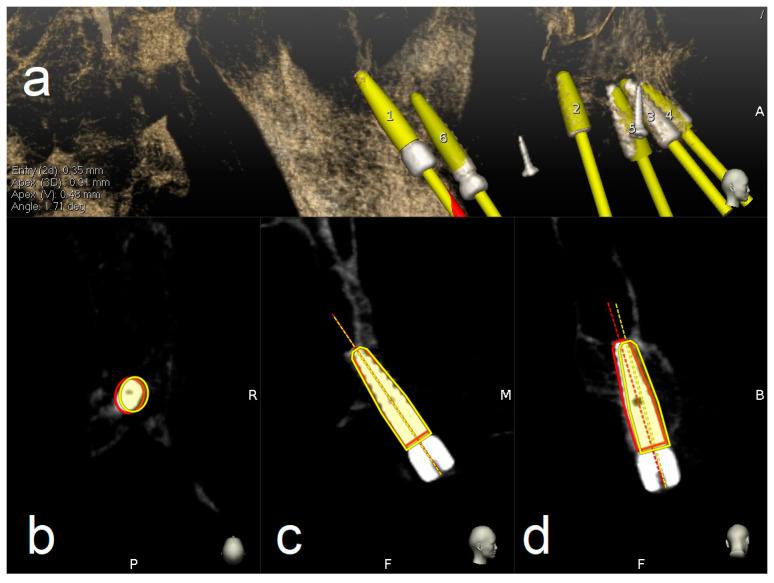
The software automatically fits an implant model to its appearance in the post-operative image (**a**) and computes the angular axis corrected between the planned and actual implant locations (implant inserted with dynamic guidance) (**b**–**d**).

The preoperative surgical plan and the postoperative CBCT were superimposed using an accuracy evaluation software (EvaluNav™) part of Navident™ navigation system (Claronav Inc., Toronto, YTO, Canada). Calibration was done directly between the two volumetric images (Figure 8). Software provides various visualization tools that confirm two CBCTs are precisely matched. Once the user is satisfied with volumetric registration, the software automatically matches the planned implant shape onto the post-operative image and computes deviations between the planned and placed implant locations (Figure 9).

To address the main goal of the study, the accuracy of pterygoid and frontal implants’ placement was assessed in terms of deviation at coronal, apical 3D, apical depth, and angular level.

Outcomes were evaluated both clinically and radiographically after three months post-loading.

**Figure 10:**
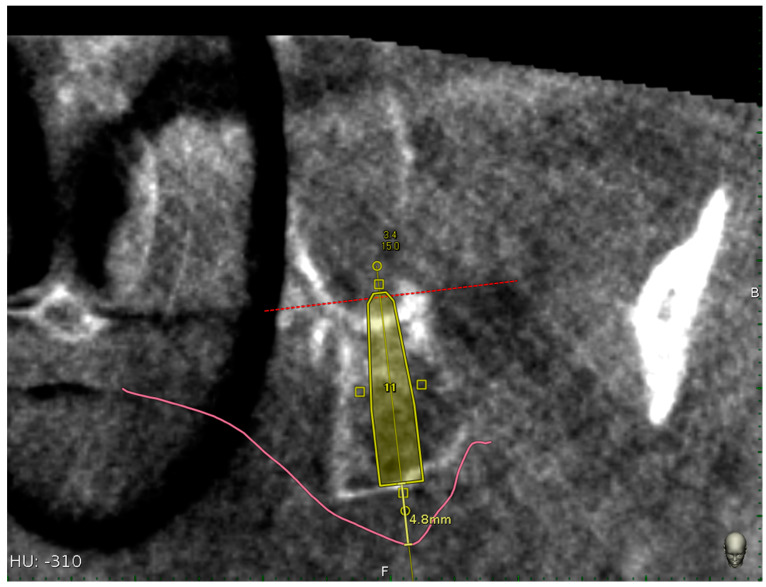
Mucosa thickness linear measurement using the implant centric view of the CBCT.

Implant survival rate, implant length, and mucosa thickness above the planned pterygoid implants were also reported (Figure 10).

The mucosa thickness was calculated as linear measurement above each planned pterygoid implants by means of implant centric CBCT view.

Furthermore, any difference in terms of passivity was evaluated using angled M.U.A. or OT-Equator abutments.

A database was created using Excel (Microsoft, Redmond, WA, USA). Descriptive statistics including mean and standard deviation values were calculated for each variable.

The independent-samples t-test was used to identify statistically significant differences in the coronal position of implants, the apical position of implants, the depth of implants, and the angle of inserted implants between pterygoid and frontal ones compared with planned implants.

Data were evaluated using standard statistical analysis software (IBM SPSS Statistics for Windows, Version 22.0. IBM Corp., Armonk, NY, USA). In each test, the cut-off for statistical significance was *p* ≤ 0.05. A power analysis using the G*Power 3.1.9.7 for Windows XP with an alpha level of 0.05 and a medium effect size (*f* = 0.90) showed that 27 implants for each group would be adequate to obtain 95% power in detecting a statistical difference between two groups in the mean.

## 3. Results

Post-operative complications were not reported by the 14 patients.

In three patients, one frontal implant failed during the first period of the immediate loading (success rate of 92.8%) and final impression was taken on the remaining five implants (two pterygoid and three frontal implants).

In one patient, a pterygoid implant, which did not have a torque useful to be immediately loaded, failed during its first month of insertion (success rate of 96.4%) and was replaced with a new one of the same length, but with a wider diameter. The provisional prosthesis was made using four frontal implants.

The average accuracy in coronal position, apical position, depth, and angle of implants compared with planned implants was reported in Table 1, Table 2 and Table 3.

Table 4 reports the average length of pterygoid implants used and Table 5 reports mucosa thickness above pterygoid implants.

The independent-samples *t*-test showed that there was a statistically significant mean difference between the frontal implants and pterygoid implants in the apical 3d (*p* = 0.001) and apical depth (*p* = 0.01) of implants; the coronal position of implants (*p* = 0.29) and angle position of implants (*p* = 0.27) showed that there was not a statistically significance between the means (Table 6).

## 4. Discussion

In this study, the placement accuracy of both pterygoid and frontal implants for a full maxillary implant supported rehabilitation (Da Vinci Bridge^®^) was assessed using a digital workflow of a dynamic computer-aided implantology system (DCAI).

DCAI works like a global positioning system by making a triangulation with the two cameras, the contra-angle handpiece, and the patient jaw. In this way, the clinician can follow on the screen in real time both the osteotomy site preparation and the implant placement.

At each step, an accuracy check will enable the clinician to prove system accuracy, and thus provide assurance that everything is set up to proceed with a safe surgery.

It is mandatory to evaluate the accuracy of these systems when an implant as well as the pterygoid one is planned to be inserted in a high risk anatomic area.

The accuracy of all 84 implants inserted using DCAI reported in this study was 0.66 mm at coronal point, 1.01 mm at apical 3D, 0.52 mm at apical depth, and 2.61° as angular deviation.

These deviation values reported in this study were consistent with those reported in the literature by other studies using the same technology or a different one with the use of a radiological stent.

Stefanelli et al., in a recent study, using TaP technology, reported, for 136 implants inserted in 59 patients, the following deviations: 0.67 mm at coronal, 0.99 at apical, 0.55 at depth, and 2.5 as angular error [34].

Block et al., using a second-generation navigation system (X-Guide™, X-Nav Technologies), compared deviations using this system versus free-hand. Three surgeons were involved and treated 100 partially edentulous patients. The results (mean (SD)) with navigation were 0.87 (0.42) mm at entry (lateral/2D), 1.56 (0.69) mm at the apex (3D), and 3.62° (2.73°) angular. The unguided deviations had corresponding means (SD) of 1.15 (0.59) mm, 2.51 (0.86) mm, and 7.69° (4.92°). No statistically significant differences were observed in navigated placement between individual clinicians [31].

Pellegrino et al. treated 10 partially or totally edentolous patients using ImplaNav™ system and reported a mean deviation value of 1.04 mm at entry point, a mean deviation value of 1.35 mm at apex, 0.45 mm as depth deviation, and 6.46° as angular deviation [37].

Stefanelli et al. reported in a retrospective observational study on 231 implants (89 arches) using Navident™ (Claronav, Toronto, YTO, Canada) an error of 0.71 mm at entry point, an error of 1 mm at apex, and a mean angular error of 2.26°. The presence of a learning curve was also evaluated. They reported an error of 0.94 mm at the entry point, 1.19 mm at apical point, and 3.48° as angular deviation in the insertion of the first 50 implants of 231, and an error of 0.59 mm at entry point, 0.85 mm at apical point, and 1.98° as angular deviation in the insertion of the last 50 implants [33].

The reported results of the accuracy using several dynamic navigation systems and several protocols are, in any case, better than the accuracy values of inserted implants by free hand (mental navigation). Vercruyssen et al. [38], analyzed accuracy by inserting dental implants using static guides or the free-hand approach (mental navigation). The deviations reported using the free-hand method were as follows: 2.8 (1.5) mm at entry point, 2.9 (1.5) mm at the apex, and 9.9° (6.0°) for angular deviations.

The deviations reported by Vercruyssen referred to the use of conventional implants (10–13 mm long); if the same angular error (10°) is done by inserting a 20 mm long pterygoid implant, the apical deviations could be 5–6 mm, and these values could be very dangerous in this high risk anatomic area.

Another evaluation of this study was to compare the accuracy between frontal and pterygoid implants to assess if the challenge to approach the pterygoid area could affect the results.

The reported deviations of the pterygoid implants were 0.72 mm at coronal point, 1.25 mm at apex 3D, 0.69 mm as apical depth, and 2.86 degree as angular error, while the deviations of frontal implants were 0.64 mm at coronal point, 0.89 mm at apical 3D, 0.46 mm as apical depth, and 2.49 mm as angular error.

A statistically significant difference was reported between means when accuracy values of the pterygoid implants were compared with frontal ones at apical 3D and apical depth; the reason for this difference was probably owing to the different implant lengths used for pterygoid implants compared with the frontal ones. These results were in accordance with the study published by Van Assche et al. [39], reporting a relationship between the length of the bur and the apical deviation.

Telasne and Tessier in 1989 [24,25] reported that 80% of native bone of the pterygoid area was preserved during the time, and this column of bone was enough to insert a 13–20 mm long implant. The pterygoid implant lengths reported by Telasne and Tessier are consistent with the results of this study, in which the range of the pterygoid implants used was from 13 to 20 mm. In particular, 15 mm (32%) and 16 mm (36%) implant lengths were the most used in this study.

Bydra et al. analyzed 897 pterygoid implants and reported a 92% index survival rate (70 implants failed before occlusal loading); they also reported failure of nine implants in post-loading phase [28].

Candel et al., in a review of the rehabilitation of the atrophic posterior maxilla with 1053 pterygoid implants in 676 patients, reported that the weighted average success of pterygoid implants was 90.7% [29].

Araujo et al., in a systematic review and meta-analysis of the literature, investigated if the pterygoid implants are predictable for the rehabilitation of atrophic posterior maxillae and which are the mean causes of their failure [30]. They reported that pterygoid implants are predictable as their 10-year survival rate is 94.85% (same rate of the conventional implants). Regarding the causes of implant failures (almost all of them were reported before the loading), the hypothesis indicated is related to the implant mispositioning owing to technique difficulties [40].

Graves et al. described a procedure to insert pterygoid implants free-hand; he suggested to insert them in the center of the pterygoid fossa where the thickness of the pterygomaxillary suture is higher; he suggested completely traversing the pterygoid plate (1–2 mm into the pterygoid fossa). A soft tissue reduction was recommended at the implant insertion stage. He inserted 63 implants and reported 7 implant failures. All the implant failures happened before loading [41].

All these authors reported a high success rate of pterygoid implants and concluded that failures were almost all in the first period before loading. Most likely, this was owing to malpositioning of implants (difficulty in a semi-blinded surgical technique) [40,42].

In this study, a higher success rate (96.4%) was reported and only one pterygoid implant failed owing to a lack of primary stability during its insertion.

This remarkable result is owing, in part, to the use of DCAI that allowed to plan and to insert an implant in an accurate position by engaging cortical plate, as planned in the CBCT with high torque values.

The mean mucosa thickness above the pterygoid implants calculated in this study was 5.08 mm (range: 2.6–8.2 mm). Referable to the amount of measured implant soft tissue, as reported by Graves [41], a soft tissue reduction is suggested at the implant placement stage.

Owing to the different implant angulation, two type of abutments were used: M.U.A. and OT Equator.

Both types of connection in all treated cases did not show any kind of problem in terms of passivity. However, OT Equator could offer several advantages. One of this is that all OT Equator are straight and their prosthetic screw is 1.3 mm, that is, 0.3 mm wider than the traditional M.U.A. screw.

Owing to screw resistance, this increment offers a 70% higher resistance to the stress value.

The limitation of this study is that the results are referred to a single surgeon in a single practice. Caution should be exercised when interpreting these results on a broader context and generalizing such results among surgeons. Additional similar in vivo accuracy studies should be undertaken to validate our results so that more data are available on a broader context in such a challenging, yet important area. Such studies would further improve the guidelines for pterygoid implants surgery protocols and optimize patient safety.

Another limitation of this study is the limited follow up of both the inserted implants and the full arch prosthesis.

## 5. Conclusions

The high accuracy values reported in this study seem to be related to the use of a dynamic navigation system.

In addition, the insertion of pterygoid implants with the use of a DCAI offers a suitable alternative to traditional reconstructive surgical needs to enable implants to be placed in the atrophic posterior maxilla, which is often deficient of vertical bone height.

## Figures and Tables

**Table 1:** Mean, maximum, and minimum of the coronal, apical 3D, apical vertical (depth), and angular deviations of the total implants inserted using a dynamic navigation system (*n* = 74).

Deviation of the Total Implant Inserted (78)	Mean	Minimum	Maximum	Standard Deviation
Coronal (mm)	0.66	0.04	2.19	0.35
Apical 3D (mm)	1.01	0.23	2.52	0.46
Apical (depth) (mm)	0.52	0.02	1.27	0.30
Angular degree (°)	2.61	0.39	7.86	1.29

**Table 2:** Mean, maximum, and minimum of the coronal, apical 3D, apical vertical (depth), and angular deviations of the pterygoid implants inserted using a dynamic navigation system (*n* = 28).

Deviation of the Pterygoid Implants Inserted (28)	Mean	Minimum	Maximum	Standard Deviation
Coronal (mm)	0.72	0.25	1.42	0.28
Apical 3D (mm)	1.25	0.56	2.52	0.46
Apical (depth) (mm)	0.69	0.06	1.27	0.33
Angular degree (°)	2.86	0.57	7.86	1.56

**Table 3:** Mean, maximum, and minimum of the coronal, apical 3D, apical vertical (depth), and angular deviations of the frontal implants inserted using a dynamic navigation system (*n* = 56).

Deviation of the Frontal Implant Inserted (56)	Mean	Minimum	Maximum	Standard Deviation
Coronal (mm)	0.64	0.04	2.19	0.37
Apical 3D (mm)	0.89	0.23	2.01	0.42
Apical (depth) (mm)	0.46	0.02	1.22	0.26
Angular degree (°)	2.49	0.39	5.21	1.14

**Table 4:** Number (%) of different implant lengths used.

Implant Length (mm)	Implants Used (%)
13	2 (7%)
15	9 (32%)
16	10 (36%)
18	3 (11%)
20	4 (14%)

**Table 5:** Mean, maximum, minimum, and standard deviation of the mucosa thickness above the pterygoid implants.

Distances (mm)	Mean	Minimum	Maximum	Standard Deviation
Mucosa thickness	5.08	2.6	8.2	1.77

**Table 6:** *t*-test showing the difference between the means of pterygoid and frontal implants. The difference was considered statistically significant for a value of *p* < 0.05.

*t*-Test (Frontal Implants vs. Navident)	Difference between Means	Sig. (*p*)
Coronal deviation (mm)	0.07	*p* = 0.29
Apical 3D (mm)	0.36	*p* = 0.001
Apical (depth) (mm)	0.20	*p* = 0.01
Angular deviation (°)	0.37	*p* = 0.27

## References

[1] Adell R., Eriksson B., Lekholm U., Branemark P.I., Jemt T. (1990). Long-term follow-up study of osseointegrated implants in the treatment of totally edentulous jaws. Int. J. Oral Maxillofac. Implant..

[2] Guarnieri R., Di Nardo D., Gaimari G., Miccoli G., Testarelli L. (2019). Short vs. Standard Laser-Microgrooved Implants Supporting Single and Splinted Crowns: A Prospective Study with 3 Years Follow-Up. J. Prosthodont..

[3] Pjetursson B.E., Tan K., Lang N.P., Bragger U., Egger M., Zwahlen M.A. (2004). Systematic review of the survival and complication rates of fixed partial dentures (FPDs) after an osservation period of at least 5 years. Clin. Oral Implant. Res..

[4] Sharan A., Madjiar D. (2008). Maxillary sinus pneumatization following extractions: A radiographic study. Int. J. Oral Maxillofac. Implant..

[5] Schropp L., Wenzel A., Kostopoulos L., Karring T. (2003). Bone healing and soft tissue contour changes following single-tooth extraction: A clinical and radiographic 12-month prospective study. Int. J. Periodontics Restor. Dent..

[6] Ellegaard B., Kolsen-Petersen J., Baelum V. (1997). Implant therapy involving maxillary sinus lift in periodontally compromised patients. Clin. Oral Implant. Res..

[7] Lollobrigida M., Fortunato L., Serafini G., Mazzucchi G., Bozzuto G., Molinari A., Serra E., Menchini F., Vozza I., De Biase A. (2020). The prevention of implant surface alterations in the treatment of peri-implantitis: Comparison of three different mechanical and physical treatments. Int J. Environ. Res. Public Health..

[8] Baggi L., Capelloni I., Di Girolamo M., Maceri F., Vairo G. (2008). The influence of implant diameter and length on stress distribution of osseointegrated implants related to crestal bone geometry: A three-dimensional finite element analysis. J. Prosthet Dent..

[9] Jaffin R.A., Berman C.L. (1991). The excessive loss of Branemark fixtures in type IV bone: A 5-year analysis. J. Periodontol..

[10] Balshi T.J., Wolfinger G.J., Slauch R.W., Balshi S.F. (2013). Brånemark system implant lengths in the pterygomaxillary region: A retrospective comparison. Implant. Dent..

[11] Fischer K., Bäckström M., Sennerby L. (2009). Immediate and early loading of oxidized tapered implants in the partially edentulous maxilla: A 1-year prospective clinical, radiographic, and resonance frequency analysis study. Clin. Implant Dent. Relat. Res..

[12] Wallace S.S., Froum S.J. (2003). Effect of maxillary sinus augmentation on the survival of endosseous dental implants. A systematic review. Ann. Periodontol..

[13] Del Fabbro M., Testori T., Francetti L., Weinstein R. (2004). Systematic review of survival rates for implants placed in the grafted maxillary sinus. Int. J. Periodontics Restor. Dent..

[14] Rose P.S., Summers R.B., Mellado J.R., Salkin L.M., Shanaman R.H., Marks M.H., Fugazzotto P.A. (1999). Bone-added osteotome sinus floor elevation technique: Multicenter retrospective report of consecutively treated patients. Int. J. Oral Maxillofac. Implant..

[15] Bahat O., Fontanessi R.V. (2001). Efficacy of implant placement after bone grafting for three-dimensional reconstruction of the posterior jaw. Int. J. Periodontics Restor. Dent..

[16] Felice P., Barausse C., Pistilli R., Ippolito D.R., Esposito M. (2018). Short implants versus longer implants in vertically augmented posterior mandibles: Result at 8 years after loading from a randomized controlled trial. Eur. J. Oral Implantol..

[17] Felice P., Barausse C., Pistilli V., Piattelli M., Ippolito D.R., Esposito M. (2018). Posterior atrophic jaws rehabilitated with prostheses supported by 6 mm long × 4 mm wide implants or by longer implants in augmented bone. 3-year post-loading results from a randomized controlled trial. Eur. J. Oral Implantol..

[18] Fan T., Li Y., Deng W.W., Wu T., Zhang W. (2017). Short implants (5–8 mm) versus longer implants (>8 mm) with sinus lifting in atrophic posterior maxilla: A meta-analysis of RCTs. Clin. Implant. Dent. Relat. Res..

[19] Anitua E., Flores J., Flores C., Alkhraisat M.H. (2016). Long-term outcomes of immediate loading of short implants: A controlled retrospective cohort study. Int. J. Oral Maxillofac. Implant..

[20] Bechara S., Kubilius R., Veronesi G., Pires J.T., Shibli J.A., Mangano F.G. (2017). Short (6-mm) dental implants versus sinus floor elevation and placement of longer (≥10 mm) dental implants: A randomized controlled trial with a 3-year follow-up. Clin. Oral Implant. Res..

[21] Chana H., Smith G., Bansal H., Zahra D. (2019). A Retrospective Cohort Study of the Survival Rate of 88 Zygomatic Implants Placed Over an 18-year Period. Int. J. Oral Maxillofac. Implant..

[22] Petrungaro P.S., Kurtzman G.M., Gonzales S., Villegas C. (2018). Zygomatic Implants for the Management of Severe Alveolar Atrophy in the Partial or Completely Edentulous Maxilla. Compend. Contin. Educ. Dent..

[23] Davó R., Felice P., Pistilli R., Barausse C., Marti-Pages C., Ferrer-Fuertes A., Ippolito D.R., Esposito M. (2018). Immediately loaded zygomatic implants vs conventional dental implants in augmented atrophic maxillae: 1-year post-loading results from a multicentre randomised controlled trial. Eur. J. Oral Implantol..

[24] Tulasne J.F., Albrektson T., Zarb G. (1989). Implant treatment of missing posterior dentition. The Brånemark Osseointegrated Implant.

[25] Tulasne J.F., Worthington P., Brånemark P.I. (1992). Osseointegrated fixtures in the pterygoid region. Advanced Osseointegration Surgery, Applications in the Maxillofacial Region.

[26] Uchida Y., Yamashita Y., Danjo A., Shibata K., Kuraoka A. (2017). Computed tomography and anatomical measurements of critical sites for endosseous implants in the pterygomaxillary region: A cadaveric study. Int. J. Oral Maxillofac. Surg..

[27] Rodríguez X., Lucas-Taulé E., Elnayef B., Altuna P., Gargallo-Albiol J., Peñarrocha Diago M., Hernandez-Alfaro F. (2016). Anatomical and radiological approach to pterygoid implants: A cross-sectional study of 202 cone beam computed tomography examinations. Int. J. Oral Maxillofac. Surg..

[28] Bidra A.S., Huynh-Ba G. (2011). Implants in the pterygoid region: A systematic review of the literature. Int. J. Oral Maxillofac. Surg..

[29] Candel E., Peñarrocha D., Peñarrocha M. (2012). Rehabilitation of the atrophic posterior maxilla with pterygoid implants: A review. J. Oral Implantol..

[30] Araujo R.Z., Santiago, Júnior J.F., Cardoso C.L., Benites Condezo A.F., Moreira Júnior R., Curi M.M. (2019). Clinical outcomes of pterygoid implants: Systematic review and meta-analysis. J. Craniomaxillofac. Surg..

[31] Block M.S., Emery R.W., Cullum D.R., Sheikh A. (2017). Implant placement is more accurate using dynamic Navigation. J. Oral Maxillofac. Surg..

[32] Mandelaris G.A., Stefanelli L.V., DeGroot B.S. (2018). Dynamic Navigation for Surgical Implant Placement: Overview of Technology, Key Concepts, and a Case Report. Compend. Contin. Educ. Dent..

[33] Stefanelli L.V., DeGroot B.S., Lipton D.I., Mandelaris G.A. (2019). Accuracy of a Dynamic Dental Implant Navigation System in a Private Practice. Int. J. Oral Maxillofac. Implant..

[34] Stefanelli L.V., Mandelaris G.A., DeGroot B.S., Gambarini G., De Angelis F., Di Carlo S. (2020). Accuracy of a novel trace registration method for dynamic navigation surgery. Int. J. Periodontics Restor. Dent..

[35] Mangano F., Veronesi G. (2018). Digital versus Analog procedures for the prosthetic restoration of single implants: A randomized controlled trial with 1 year of follow-up. Biomed. Res. Int..

[36] Mangano F.G., Hauschild U., Admakin O. (2018). Full in-Office guided surgery with open selective tooth-supported templates: A prospective clinical study on 20 patients. Int. J. Environ. Res. Public Health.

[37] Pellegrino G., Taraschi V., Andrea Z., Ferri A., Marchetti C. (2019). Dynamic navigation: A prospective clinical trial to evaluate the accuracy of implant placement. Int. J. Comput. Dent..

[38] Vercruyssen M., Cox C., Coucke W., Naert I., Jacobs R., Quirynen M. (2014). A randomized clinical trial comparing guided implant surgery (bone- or mucosa-supported) with mental navigation or the use of a pilot-drill template. J. Clin. Periodontol..

[39] Van Assche N., Quirynen M. (2010). Tolerance within a surgical guide. Clin. Oral Implant. Res..

[40] Park Y.J., Cho S.A. (2010). Retrospective chart analysis on survival rate of fixtures installed at the tuberosity bone for cases with missing unilateral upper molars: A study of 7 cases. J. Oral Maxillofac. Surg..

[41] Graves S.L. (1994). The pterygoid plate implant: A solution for restoring the posterior maxilla. Int. J. Periodontics Restor. Dent..

[42] Salinas-Goodier C., Rojo R., Murillo-González J., Prados-Frutos J.C. (2019). Three-dimensional descriptive study of the pterygomaxillary region related to pterygoid implants: A retrospective study. Sci. Rep..

